# Supplementary figures and images for: The Relationship Between Soil and Gut Microbiota Influences the Adaptive Strategies of Goitered Gazelles in the Qaidam Basin
Source: Animals (Basel). 2024 Dec 15;14(24):3621. doi: 10.3390/ani14243621 (PMC11672517; doi:10.3390/ani14243621)

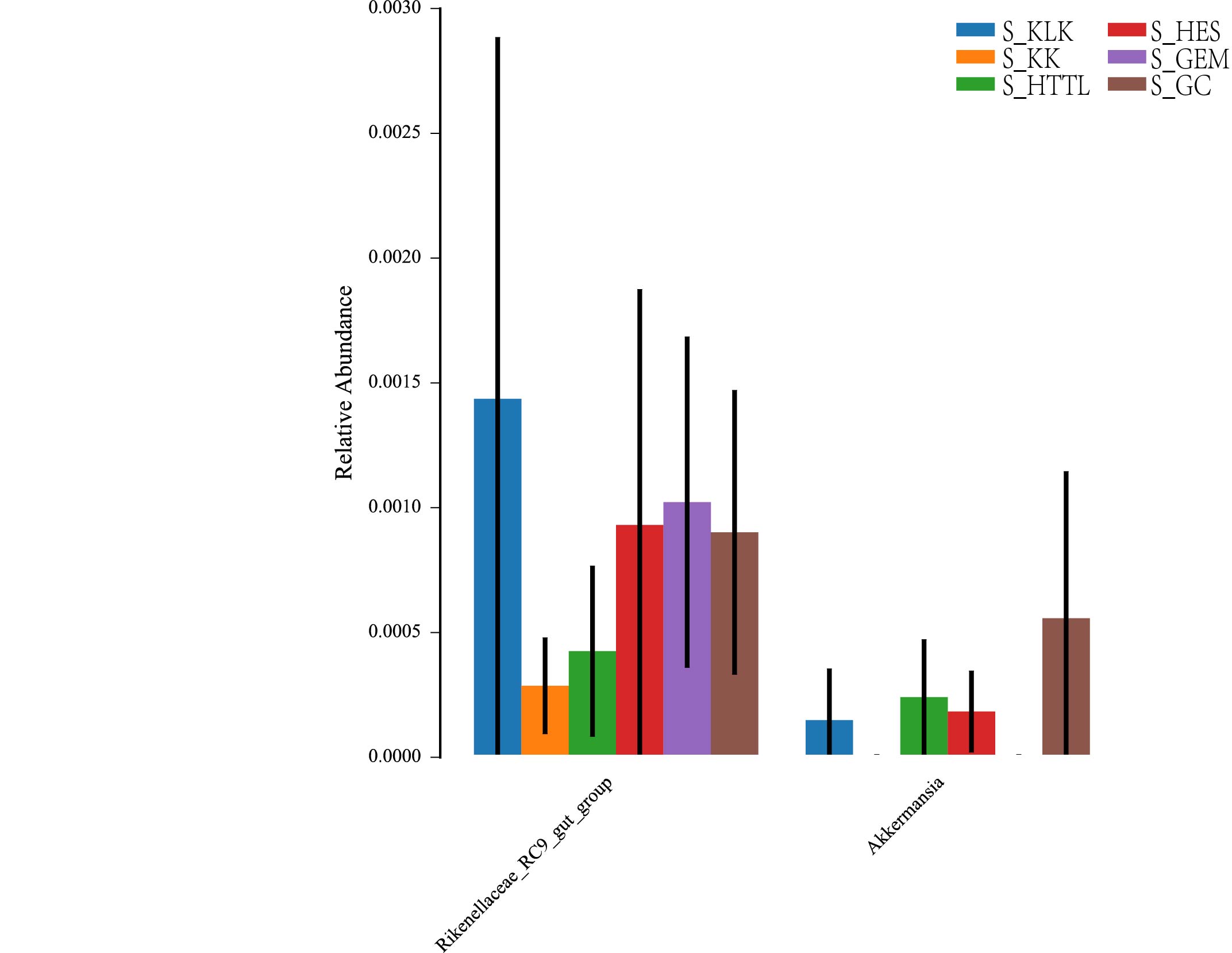

Supplement: Supplementary file 1 [file animals-14-03621-s001.zip › supplementary material/supplementary material/Supplementary Figure 1 Relevant genus-level information about bacteria in soil.jpg]
